# Clustering of rheumatoid arthritis patients: an unsupervised machine learning approach for characterizing the TNFi response

**DOI:** 10.1007/s12026-026-09822-x

**Published:** 2026-08-17

**Authors:** Pedro Augusto Silva dos Santos Rodrigues, Katarina Mattos Brandão, Lilian de Sá Garcia Landeiro, Laryssa Cardoso Calmon, Almirane Lima de Oliveira, Thamara Barbosa Miranda, João Locke Ferreira de Araújo, Rafael Valente Veiga, Camila Alexandrina Viana de Figueiredo, Pablo de Moura Santos, Ryan dos Santos Costa

**Affiliations:** 1 Department of Bioregulation, Laboratory of Immunopharmacology and Molecular Biology, Institute of Health Sciences, Federal University of Bahia, UFBA, Avenue Reitor Miguel Calmon, Canela, Salvador, BA 40110-100 Brazil; 2 Department of Medicine, School of Pharmacy, Federal University of Bahia, Salvador, Bahia Brazil; 3 Center for Infusions and Specialized Medicines of Bahia (CIMEB), Directorate of Pharmaceutical Assistance, Health Secretary of the State of Bahia, Salvador, Bahia Brazil

**Keywords:** Cluster analysis, Tumor necrosis factor inhibitors, Rheumatoid arthritis, Multivariate analysis, Precision medicine

## Abstract

This study aimed to identifydistinct profiles of response to Tumor Necrosis Factor inhibitors (TNFi) in rheumatoid arthritis (RA) patients using unsupervised machine learning to integrate clinical, demographic, and genetic data. A cohort of 294 RA patients was analyzed using hierarchical clustering techniques. Data collected included body mass index (BMI), adherence to physical activity, prevalence of comorbidities, seropositivity, Health Assessment Questionnaire (HAQ) scores, and genetic polymorphisms in TNF pathway. Responders to TNFi demonstrated a more favorable clinical profile, with a lower BMI (25.6 vs. 27.0; *p* = 0.03), higher physical activity adherence (56.6% vs. 33.3%; *p* < 0.01), reduced prevalence of comorbidities (69.8% vs. 82.4%; *p* = 0.02), and improved functionality (HAQ: 0.8 vs. 1.7; *p* < 0.01) compared with non-responders. The C allele of rs767455 was significantly more frequent among responders (74.4% vs. 62.4%; *p* = 0.04). Clustering analysis identified three distinct subgroups: a better prognosis group characterized by a 73.5% response rate, minimal use of combination therapies, high adherence to physical activity, and male predominance; a poorer therapeutic response group with 82.9% therapeutic failure, intensive medication use, and low exercise adherence; and an intermediate group. TNFi response in RA is multifactorial. Integrating clinical, demographic, and genetic data enables the identification of distinct patient profiles, supporting the development of personalized treatment strategies and the advancement of precision medicine in RA management.

**Supplementary Information:**

The online version contains supplementary material available at 10.1007/s12026-026-09822-x.

## Introduction

Rheumatoid arthritis (RA) is an autoimmune disease characterized by chronic inflammation of the synovial tissue in multiple joints, leading to irreversible joint destruction, deformities, and significant impairment of patients’ quality of life [1]. The prevalence of RA in Latin America is not well explored, but studies estimate a prevalence of 0.46%, with most diagnosed patients being women aged between 30 and 50 years [2, 3].

RA development is triggered by genetic, sociodemographic, and environmental factors, including infectious agents, hormones, and diet, with obesity and smoking being the most well-studied associations [4–6]. The laboratory profile of RA is also altered due to the presence of autoantibodies and other immune mechanisms that influence chemokine and cytokine secretion, contributing to increased immune response, chronic inflammation, and bone destruction [6].

Tumor necrosis factor inhibitors (TNFi) play a crucial role in RA treatment. TNF is a pro-inflammatory cytokine found in high concentrations in the serum and synovial fluid of RA patients, contributing to bone resorption and inducing other pro-inflammatory cytokines, such as IL-6, which perpetuate the inflammatory condition [7, 8]. Although TNFi effectively improves symptoms and reduces mortality, 30% to 40% of patients experience an insufficient response or loss of response within a year [9, 10]. Identifying patterns related to TNFi response can optimize treatment selection and improve therapeutic efficacy.

A previous study on a cohort of RA patients treated with TNFi demonstrated that genetic variants in the TNF pathway are involved in treatment response and laboratory alterations in these patients [11]. Using unsupervised machine learning methods, this study aimed to explore phenotypic patterns of rheumatoid arthritis. The application of these techniques may offer a deeper understanding of clinical phenotypes and genetic factors associated with treatment, guiding clinical practice and future research.

## Materials and methods

### Study design and population

This study is part of an ambispective cohort that included 294 patients diagnosed with RA and undergoing TNFi treatment, registered in the Specialized Pharmaceutical Assistance Component of Bahia, Brazil. All patients provided informed consent. The follow-up period lasted from January 2019 to June 2023. Patient recruitment occurred between 2019 and 2023; however, recruitment was paused from March 2020 to July 2021 due to the COVID-19 pandemic. The retrospective component comprised reconstruction of pre-recruitment clinical and treatment history using medical records and patient reports (e.g., prior medications, adverse events, and therapeutic failures). The prospective component comprised post-recruitment follow-up with standardized reassessment of outcomes, including a second HAQ application at ≥ 6 months after recruitment.

Inclusion criteria consisted of adult patients (≥ 18 years) diagnosed with RA according to the Clinical Protocol and Therapeutic Guidelines established by the Brazilian Ministry of Health [12], registered in the Specialized Pharmaceutical Assistance Component of Bahia and receiving TNFi at the infusion centers where the research was conducted. Exclusion criteria included failure to meet the inclusion criteria, non-adherence to treatment, diagnostic uncertainty, and death during the study period. Patients with other autoimmune diseases were excluded. Although severe infections, malignancies, and other major clinical conditions were considered exclusion criteria, no participants were excluded for these reasons in the final cohort. Additionally, because the infusion centers where the research was conducted are outpatient services, patients with severe or uncontrolled comorbidities (e.g., severe infections, malignancies, or severe cardiovascular diseases) are not managed at this setting and therefore were not part of the source population. Previous exposure to other biologic DMARDs was not an exclusion criterion, because the study aimed to reflect real-world TNFi-treated patients with heterogeneous treatment histories; therefore, prior biologic exposure and therapy changes were collected and summarized. This study was approved by the Research Ethics Committee of the Professor Edgard Santos University Hospital – HUPES (CAAE: 89660418.8.0000.0049 / Opinion No. 5.341.882).

### Data collection

Data were obtained through telephone interviews, electronic medical records, printed (paper) medical records, and medication dispensing records. Collected information included demographic profile (sex, age, BMI, race/ethnicity), clinical history (comorbidities, time since diagnosis, and age at diagnosis), medication use (synthetic DMARDs, biological DMARDs, other medications, adverse events, and therapy changes), and treatment response assessed using a Brazilian validated Portuguese version of the Health Assessment Questionnaire (HAQ) and the Visual Analog Scale (VAS). Ethnicity was self-reported according to categories commonly used in the Brazilian population, and the “multiethnic” category reflects the highly admixed demographic composition characteristic of the Brazilian population.

Physical activity was assessed by self-report during clinical evaluation using a categorical variable (ATIV_FIS), and participants were classified as physically active or non-active for comparative and clustering analyses. The variable “presence of at least one comorbidity” was defined as the presence of one or more chronic clinical conditions reported during clinical evaluation or identified in medical records, including hypertension, diabetes mellitus, dyslipidemia, respiratory diseases, neoplasms, and related conditions.

Additional medications referred to non-biologic supportive therapies and medications used for symptom control or management of associated conditions, including analgesics, nonsteroidal anti-inflammatory drugs, corticosteroids, and other general medications recorded during clinical evaluation.

Although related, the variables “presence of at least one comorbidity” and “use of additional medications” were analyzed separately because they reflect distinct clinical dimensions, namely disease burden and therapeutic complexity/supportive pharmacological management, respectively.

The questionnaires were applied at two time points: at recruitment and again after a mean follow-up interval of 9.5 ± 8.6 months for all participants. Variability in follow-up intervals was partially influenced by restrictions associated with the COVID-19 pandemic and by differences in ongoing treatment duration at study inclusion. For the purposes of this study, response to the TNFi in use during follow-up was primarily assessed using the HAQ obtained at the follow-up interview (i.e., the last HAQ assessment), since ≥ 6 months is sufficient to capture an initial treatment response among patients initiating therapy at baseline. The HAQ is scored on a scale ranging from 0 to 3, where a score of 0 reflects optimal functional capacity and a score of 3 indicates maximal functional impairment [13, 14].

For the assessment of TNFi treatment response, patients were classified according to both their functional status at the time of recruitment and their therapeutic history. Patients were classified as responders if they were receiving a TNFi at the time of evaluation and presented a Health Assessment Questionnaire (HAQ) score ≤ 1.5, indicating favorable functional status under the current treatment. Patients were classified as non-responders if they had a HAQ score > 1.5 despite ongoing TNFi therapy [15] or had a documented history of TNFi discontinuation due to therapeutic failure. Thus, the non-responder group included both patients with an unfavorable functional outcome during current treatment and those with previous TNFi treatment failure requiring therapy change.

### Genotyping

Peripheral blood was collected on the day of patient recruitment for genetic analyses. DNA was extracted from peripheral blood samples using the Gentra Puregene Blood Kit (QIAGEN). Genotyping was performed using TaqMan probes on the QuantStudio 12 K Flex system (Applied Biosystems). The analyzed SNPs included: rs1800629 (TNFA); rs1061622 and rs1061624 (TNFRSF1B); rs4149570 and rs767455 (TNFRSF1A). Further details on laboratory analyses can be found in a previous publication [11].

### Data preparation

Data processing was performed in R 4.3.0. The initial database comprised 30 variables. Variables with ≥ 10% missingness were excluded, and remaining missing data were imputed using MICE (Multiple Imputation by Chained Equations) for continuous variables and missForest for categorical variables [16]. To reduce computational burden and improve convergence, continuous variables were imputed in smaller subsets and subsequently merged using the unique patient identifier. Post-imputation quality control assessed whether summary distributions and key group contrasts were preserved, using Shapiro–Wilk testing followed by t-test or Wilcoxon test for continuous variables and chi-square or Fisher’s exact test for categorical variables. Continuous variables were min–max normalized to the [0,1] range. Multinomial categorical variables were converted into binary dummy variables.

### Multiple Correspondence Analysis (MCA)

MCA was performed to gain a broader understanding of relationships between categorical variables. This technique analyzes a set of observations distributed in Euclidean space, helping identify patterns through geometric methods [17]. Interpretation is based on the distances between data points, represented as coordinates on a simplified perceptual map, providing crucial insights into variable relationships [18]. MCA was applied to the selected categorical/dummy variables to obtain low-dimensional coordinates for each individual, and these MCA coordinates were carried forward to the clustering step as part of the clustering input.

### Principal Component Analysis (PCA) and factor analysis

To better understand the spectrum of response phenotypes in RA patients treated with TNFi, we applied Principal Component Analysis (PCA) combined with Factor Analysis (FA) [19]. These methods align highly correlated continuous variables into principal components, concentrating most of the information from the initial variable set into the first principal components (PCs), thereby aiding in dimensionality reduction. PC selection was based on eigenvalues > 1, following Kaiser’s criterion. Bartlett’s test of sphericity confirmed data suitability [20].

Factor analysis was then performed using the extracted PCs to identify latent factors explaining relationships among observed variables. A ranking system was created based on factor scores, weighted by the variance explained by each factor [20]. Factor scores (from the retained components) were used as quantitative summaries of continuous-variable structure and were combined with MCA-derived coordinates to form the final feature space used for clustering.

### Clustering analysis

Two clustering methods were applied to determine the best model: hierarchical clustering and k-means. The input for clustering consisted of results from MCA and Factor Analysis [20, 21]. In hierarchical clustering, the average-linkage method was used to group patients based on clinical, sociodemographic, and genetic characteristics. The optimal number of clusters was determined by maximizing the average silhouette coefficient, while cluster quality was assessed using the Dunn index, Jaccard index, and intracluster inertia. The final clustering method was selected based on model quality indicators. In addition, cluster stability was evaluated via bootstrap resampling using a Jaccard similarity approach (clusterboot procedure; B = 100), with higher Jaccard values indicating more stable/reproducible clusters. This stability assessment was used to support the choice of the final clustering solution. The two-dimensional representations were used as exploratory visualization tools to facilitate interpretation of similarity patterns and cluster organization. Dunn and Jaccard indices were used to assess cluster compactness, separation, and stability, supporting the structural consistency of the clustering solution rather than serving as direct validation of the biological significance of the two-dimensional projections [22, 23].

**Fig. 1:**
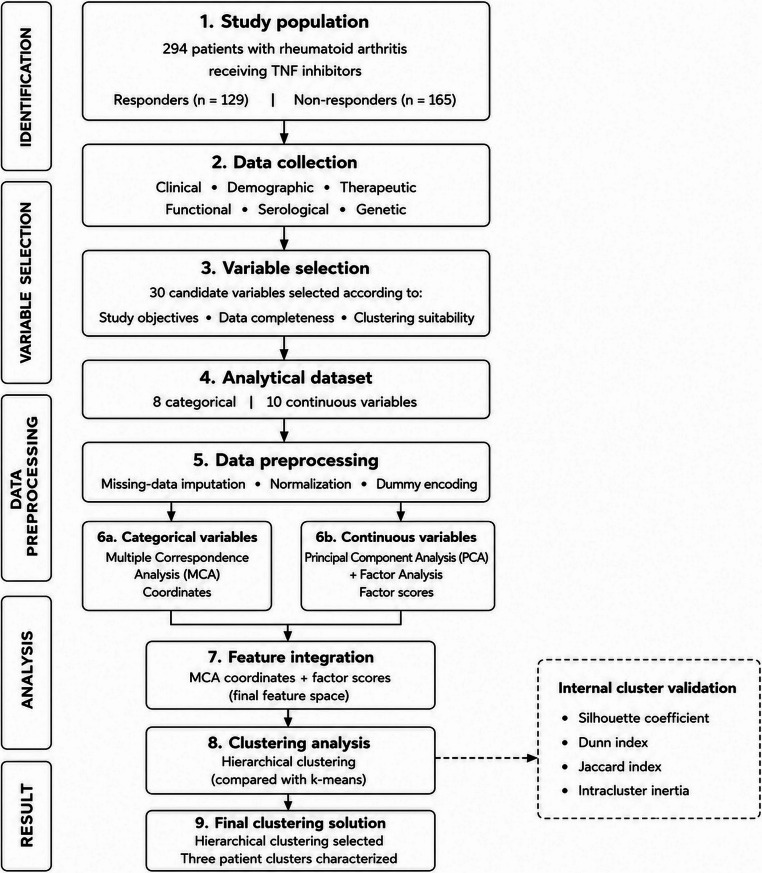
Overall study design and analytical workflow. Schematic representation of the study workflow, including patient recruitment, data collection, data preprocessing, dimensionality reduction, clustering analysis, model evaluation, and characterization of the identified patient clusters

The overall study design and analytical workflow are summarized in Fig. 1.

## Results

### Data characterization and population description

The demographic analysis revealed significant differences between responders and non-responders to TNFi treatment. A higher proportion of women was observed among non-responders (97.6%) compared to responders (86.0%), with *p* < 0.01. In contrast, no significant differences were found in age (*p* = 0.58) or ethnic distribution between the groups (*p* = 0.30). These results are presented in Table 1.

Additionally, lifestyle factors played an important role, as a higher percentage of responders engaged in physical activity (56.6%) compared to non-responders (33.3%) (*p* < 0.01). Body mass index (BMI) was also significantly lower in responders (25.6 ± 4.7) compared to non-responders (27.0 ± 5.3) (*p* = 0.03), potentially reflecting the association between obesity and reduced TNFi effectiveness.

Regarding clinical conditions, non-responders had a higher prevalence of comorbidities (82.4% vs. 69.8%, *p* = 0.02) and required additional medications more frequently (93.9% vs. 86.0%, *p* = 0.04), indicating greater clinical complexity in this group (Table 2). Seropositivity was more prevalent among responders (79.1%) compared to non-responders (58.8%) (*p* < 0.01), suggesting that patients with a well-defined immunological profile respond better to TNFi. In terms of functional impact, responders exhibited better functional capacity (HAQ: 0.8 ± 0.6 vs. 1.7 ± 0.7, *p* < 0.01) and lower pain intensity (VAS: 4.4 ± 3.1 vs. 6.4 ± 2.8, *p* < 0.01).

**Table 1:** Characterization of the study population based on response to TNFi treatment in rheumatoid arthritis

Phenotype	TNFi responder (*n* = 129)	TNFi non-responder (*n* = 165)	Significance (*p*-value)
Sex - *n* (%)			
Female	111 (86.0%)	161 (97.6%)	< 0.01**
Male	18 (14.0%)	4 (2.4%)	
Current age (years)	56.2 ± 11.8	55.2 ± 13.1	0.49
Self-reported ethnicity - n (%)			0.30
Multiethnic	69 (53.5%)	87 (52.7%)	
Black	39 (30.2%)	60 (36.4%)	
White	21 (16.3%)	18 (10.9%)	
Physical activity practive – n (%)			
No	56 (43.4%)	110 (66.7%)	< 0.01**
Yes	73 (56.6%)	55 (33.3%)	
BMI (kg/m²)	25.6 ± 4.7	27.0 ± 5.3	0.03*
Smoking – n (%)			
Non-Smokers	99 (76.7%)	124 (75.2%)	0.33
Former smokers	28 (21.7%)	33 (20.0%)	
Smokers	2 (1.6%)	8 (4.8%)	
Use of medications (except treatment for RA) - n (%)			0.04*
No	18 (14.0%)	10 (6.1%)	
Yes	111 (86.0%)	155 (93.9%)	
Presence of at least one comorbidity - n (%)			
No	39 (30.2%)	29 (17.6%)	0.02*
Yes	90 (69.8%)	136 (82.4%)	
Time since RA diagnosis (years)	15.1 ± 10.0	16.8 ± 10.4	0.09
Age at RA diagnosis (years)	40.5 ± 15.4	38.8 ± 12.8	0.33
RA classification based on the presence of autoantibodies			
Seronegative	16 (12.4%)	21 (12.7%)	< 0.01**
Seropositive	102 (79.1%)	97 (58.8%)	
Unspecified	11 (8.5%)	47 (28.5%)	
Use of non-biologic medications for RA – n (%)			
No	24 (18.6%)	46 (27.9%)	0.09
Yes	105 (81.4%)	119 (72.1%)	
bDMARD substitutions (per individual)	0.4 ± 0.6	1.6 ± 0.7	< 0.01**
Number of TNFi used (per individual)	1.3 ± 0.6	1.5 ± 0.8	< 0.01**
Adverse events related to TNFi use – n (%)			
No	106 (82.2%)	126 (76.4%)	0.29
Yes	23 (17.8%)	39 (23.6%)	
Leukocytes (x 10³ cells/mm³)	6.5 ± 2.1	6.3 ± 2.4	0.27
Platelets (x 10³ units/mm³)	259.1 ± 68.2	255.2 ± 81.2	0.14
HAQ	0.8 ± 0.6	1.7 ± 0.7	< 0.01**
VAS	4.4 ± 3.1	6.4 ± 2.8	< 0.01**

Stratification was conducted according on response to the TNFi. Normality of variables was assessed using the Shapiro-Wilk test. For categorical variables, the chi-square test was applied. Continuous variables with non-normal distribution were analyzed using the Mann-Whitney U test, while those with normal distribution were evaluated using the t-test. Continuous data are presented as mean ± standard deviation (SD). The value of n represents the subtotal of individuals per group. A p-value < 0.05 was considered statistically significant. SD: standard deviation; p: p-value, * <0.05; **<0.01

After data preprocessing, the final analytical dataset for multivariate analyses included 18 variables, consisting of 8 categorical and 10 continuous variables. A total of 14 variables with missing values underwent data imputation, as presented in Supplementary Table 1.

The stratification of variants in genes within the TNF pathway revealed that the C allele of rs767455 was more prevalent among responders (74.4%) than non-responders (62.4%), with a statistically significant difference (*p* = 0.04), suggesting a possible association of this polymorphism with treatment effectiveness. The presence of polymorphic alleles in the rs1800629, rs1061622, and rs4149570 variants was not associated with any specific response profile (Supplementary Table 2).

### Comparison between clusters obtained by *k*-means and hierarchical clustering

Based on the average silhouette coefficient, the optimal number of clusters was set to three (k = 3). To assess the quality of the groupings, the results from each clustering method were compared using different quality indicators, as presented in Table 2.

**Table 2:** Comparison of quality indicators between clustering methods

Cluster	Silhouette Coefficient	Intracluster Inertia	Dunn Index	Jaccard Index
Cluster K	0.37	72.3	0.04	K1: 0.68 K2: 0.29 K3: 0.53
Cluster H	0.37	78.1	0.09	H1: 0.84 H2: 0.91 H3: 0.94

Evaluation metrics for the clusters generated by the k-means (Cluster K) and hierarchical (Cluster H) methods. The silhouette coefficient measures cluster cohesion. Intracluster inertia was summarized using a within-cluster sum of squares measure computed for both clustering solutions to allow comparability. The Dunn index assesses separation between clusters relative to within-cluster dispersion. Cluster stability was evaluated using a bootstrap-based Jaccard index, with higher values indicating more stable cluster assignments

**Fig. 2:**
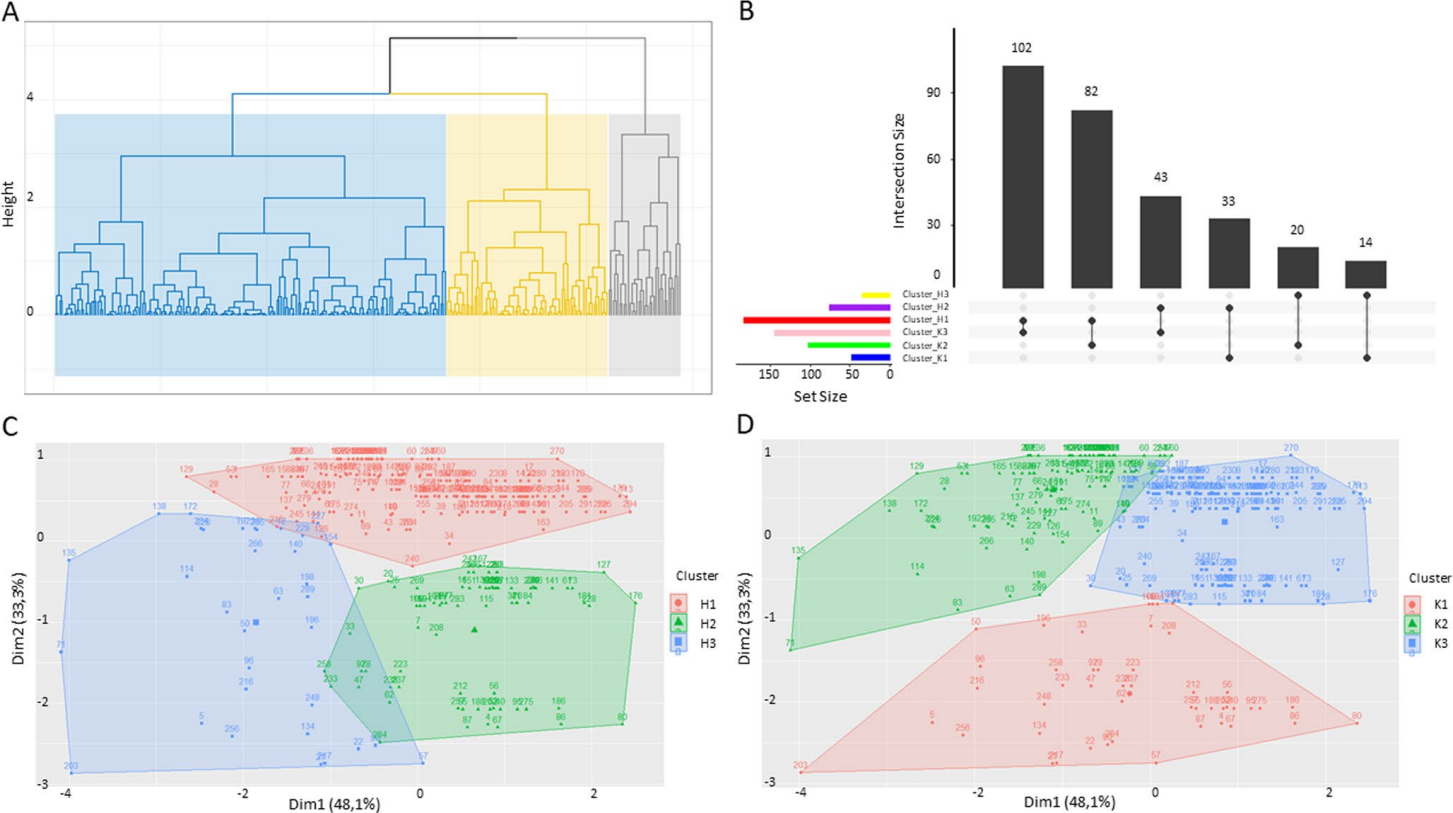
Comparison of clustering methods. **A** Hierarchical Clustering Dendrogram, where each color represents a distinct grouping identified by the hierarchical method. **B** UpSet Plot comparing the distribution of individuals among the clusters formed by the hierarchical and k-means methods, highlighting the correspondence between the segmentations. **C** Cluster Separation Plot of the Hierarchical Clustering, demonstrating the distribution of individuals according to the generated groups. **D** Cluster Separation Plot of the K-Means, presenting the segmentation of individuals based on the k-means algorithm

The silhouette coefficient for both clustering methods was approximately the same (0.37), indicating similar cohesion within the formed clusters. Regarding intracluster inertia, hierarchical clustering (78.1) showed a higher value than *k*-means (72.3), suggesting greater data dispersion within hierarchical clusters. High inertia values can also indicate lower internal cohesion among groups.

The Dunn index, which measures both separation between clusters and internal cohesion, was higher in hierarchical clustering (0.09) compared to *k*-means (0.04). This suggests that the hierarchical clustering method generated clusters that were better separated and had less overlap.

Regarding the Jaccard index, cluster stability assessed via bootstrap resampling (clusterboot) showed better values across all clusters in hierarchical clustering (H1: 0.84, H2: 0.91, H3: 0.94) compared to *k*-means (K1: 0.68, K2: 0.29, K3: 0.53). This indicates that the hierarchical clustering method achieved a more consistent grouping structure, with less noise or ambiguity in element assignments.

These results suggest that hierarchical clustering provided a more structurally consistent grouping solution, as evidenced by the higher Dunn and Jaccard indices. Although the higher intracluster inertia value could indicate more dispersed clusters, the combined indicators demonstrated that hierarchical clustering outperformed *k*-means for the dataset used, yielding a more well-defined and less overlapping segmentation. Although separation indices were modest, bootstrap resampling indicated high cluster stability, supporting that the identified structure is reproducible rather than noise. It is important to note that the visual overlap observed in Fig. 2 reflects a two-dimensional representation used for visualization and may not fully preserve separation in the clustering feature space; therefore, cluster separation and stability were primarily interpreted based on the quantitative indices computed from the clustering inputs. The dendrogram and comparisons of individual distributions within each clustering method are shown in Fig. 2.

### Evaluation of variables based on hierarchical clustering results

Hierarchical clustering revealed distinct profiles among rheumatoid arthritis patients, differentiated by treatment response, use of additional medications for comorbidities, physical activity levels, genetic characteristics, and the presence of comorbidities (Table 3).

Cluster 3 stands out as the group with the highest treatment success rate with TNFi (73.5%), the lowest dependence on additional medications (70.6% do not use them), and the highest prevalence of physically active individuals (61.8%). Additionally, this cluster includes a significantly higher proportion of men (38.2%) and a lower burden of comorbidities (17.7%), suggesting a healthier and more treatment-responsive profile.

**Table 3:** Grouping of categorical variables by hierarchical clustering

Variable		1	2	3	X² (*p* values)
TNFi response	No	91 (49.5%)	13 (17.1%)	25 (73.5%)	36.6 (< 0.01)**
	Yes	93 (50.5%)	63 (82.9%)	9 (26.5%)	
Use of other drugs	No	4 (2.2%)	0 (0.0%)	24 (70.6%)	166.7 (< 0.01)**
	Yes	180 (97.8%)	76 (100.0%)	10 (29.4%)	
Physical activity	No	100 (54.4%)	53 (69.7%)	13 (38.2%)	10.4 (< 0.01)**
	Yes	84 (45.7%)	23 (30.3%)	21 (61.8%)	
Sex	Female	178 (96.7%)	73 (96.0%)	21 (61.8%)	52.6 (< 0.01)**
	Male	6 (3.3%)	3 (4.0%)	13 (38.2%)	
rs767455_T	No	15 (8.2%)	19 (25.0%)	3 (8.8%)	14.4 (< 0.01)**
	Yes	169 (91.8%)	57 (75.0%)	31 (91.2%)	
rs1061624_A	No	59 (32.1%)	17 (22.4%)	4 (11.8%)	7.2 (0.03)*
	Yes	125 (67.9%)	59 (77.6%)	30 (88.2%)	
Presence of at least one comorbidity - n (%)	No	30 (16.3%)	10 (13.2%)	28 (82.4%)	76.1 (< 0.01)**
	Yes	154 (83.7%)	66 (86.8%)	6 (17.6%)	
RA Classification	Seronegative	0 (0.0%)	26 (34.2%)	11 (32.4%)	70.9 (< 0.01)**
	Seropositive	183 (99.5%)	0 (0.0%)	16 (47.0%)	250.7 (< 0.01)**
	Unspecified	1 (0.5%)	50 (65.8%)	7 (20.6%)	144.6 (< 0.01)**

The significant results of the categorical data indicate the total number of individuals in the cluster and the percentage that this total represents of the study population. *X*^*2*^: chi-square; F: F value; p: p-value. * <0.05; **<0.01

On the other hand, Cluster 2 has the highest TNFi failure rate (82.9%), a high dependence on multiple therapies (100.0% of individuals use other medications), and low adherence to physical activity (30.3%). This group also shows a higher proportion of patients with comorbidities (86.8%) and a lower frequency of the T allele of the rs767455 polymorphism (75.0%) compared to the other clusters, suggesting a profile of more vulnerable patients who are less responsive to treatment. In contrast, Cluster 1 presents intermediate characteristics, with approximately a 50.0% response rate to TNFi, a high prevalence of patients with comorbidities (83.7%), and a predominance of women (96.7%), being mainly composed of seropositive individuals for rheumatoid arthritis (99.5%). The high proportion of unspecified autoantibody records observed in Cluster 2 likely reflects heterogeneity and incompleteness in retrospective laboratory documentation obtained from real-world clinical records rather than a biologically distinct serological profile.

Genetic analysis also revealed significant differences among the groups, with a higher frequency of the A allele of the rs1061624 polymorphism in Cluster 3 (88.2%), which may be associated with a better response profile. These findings suggest that Cluster 3 represents a group with a better prognosis, lower need for combined therapies, and greater treatment response, while Cluster 2 includes patients with poorer TNFi response profiles and greater clinical impairment. These results are presented in Table 3 and Supplementary Table 3.

The analysis of numerical variables, shown in Table 4, further supports the functional differences among the clusters, particularly concerning TNFi response. The HAQ score was significantly lower in Cluster 3 (0.8 ± 0.7, *p* = 0.04), indicating a lower disease impact and a better prognosis for the patients in this cluster. In contrast, Cluster 2 had the worst score (1.5 ± 0.7), aligning with its high TNFi failure rate and greater comorbidity burden. Cluster 1 exhibited an intermediate profile (1.32 ± 0.79).

**Table 4:** Grouping of numerical variables by hierarchical clustering

Variable	1	2	3	F (*p* values)
Current age	56.2 ± 11.4	57.3 ± 12.9	50.0 ± 15.0	3.4 (0.06)
Age at diagnosis	40.0 ± 13.8	40.0 ± 13.4	35.9 ± 16.4	1.6 (0.20)
Time since diagnosis	16.2 ± 10.4	16.3 ± 10.2	14.5 ± 9.6	0.5 (0.47)
BMI	26.5 ± 5.1	26.5 ± 5.2	25.4 ± 4.3	0.9 (0.36)
VAS	5.6 ± 3.2	5.9 ± 2.8	4.2 ± 2.9	2.3 (0.13)
HAQ	1.3 ± 0.8	1.5 ± 0.7	0.8 ± 0.7	4.3 (0.04)*
Leukocytes (x 10³ cells/mm³)	6.4 ± 2.4	6.3 ± 2.2	6.9 ± 2.1	0.7 (0.41)
Platelets (x 10³ units/mm³)	258.9 ± 75.3	246.8 ± 72.4	268.8 ± 83.9	0.0 (0.96)
Number of changes in biological treatment	1.0 ± 0.9	1.4 ± 0.8	0.6 ± 0.7	0.6 (0.44)
Number of TNFi used	1.4 ± 0.8	1.4 ± 0.7	1.4 ± 0.8	0.6 (0.44)

The numerical data results indicate the mean ± standard deviation of the variable in the cluster. F: F value; p: p-value. * <0.05; **<0.01; *** <0.001

## Discussion

This is the first Brazilian study to employ unsupervised machine learning tools to evaluate the response profile to TNFi drugs in patients with rheumatoid arthritis. The integration of machine learning techniques with clinical, demographic, and genetic data enabled the identification of distinct profiles, demonstrating that combining multiple data dimensions can reveal potentially clinically relevant patterns of heterogeneity in TNFi effectiveness and help generate hypotheses to support more personalized clinical decision-making [22].

The application of unsupervised methods, such as multiple correspondence analysis, principal component analysis, and clustering, allowed for mapping patient heterogeneity. The identification of different clusters, reflecting variations in demographic factors, lifestyle, and genetic aspects, highlights that treatment response is not determined by a single factor but rather by a complex interaction between multiple elements [23, 24]. This finding reinforces the need for multidimensional approaches in patient stratification and the implementation of more targeted therapeutic interventions. Importantly, although separation indices were modest, bootstrap resampling indicated high cluster stability, supporting that the identified structure is reproducible rather than noise.

The results also emphasize the influence of non-immunological factors on TNFi response, such as sex differences, physical activity, and body mass index. Evidence suggests that female patients exhibit a lower response to TNFi compared to male patients, possibly due to the influence of sex hormones and differences in drug metabolism [25]. The marked female predominance observed in the cohort is consistent with the established epidemiology of rheumatoid arthritis, which disproportionately affects women, particularly in specialized referral centers managing moderate-to-severe disease [3].

Additionally, physical activity and a lower BMI are associated with improved clinical outcomes. These observations align with recent findings indicating that adiposity may negatively impact TNFi bioavailability and effectiveness, highlighting a potential role of physical exercise as a modulator of systemic inflammation [26]. In addition to inflammatory modulation, obesity may influence TNFi pharmacokinetics through altered drug distribution and increased inflammatory adipokine production, potentially reducing therapeutic effectiveness. Conversely, physical activity has been associated with improved functional capacity, reduced fatigue, and better overall disease control in patients with rheumatoid arthritis [27]. These findings reinforce the idea that metabolic and behavioural factors may interact with immunological pathways and contribute to variability in treatment response [28–30].

These results, which correlate with treatment effectiveness, suggest that lifestyle interventions may potentially modulate clinical outcomes. This perspective broadens the understanding that, beyond biological mechanisms, behavioural and social factors play a crucial role in the management of rheumatoid arthritis. The higher prevalence of comorbidities and multiple medication use among non-responders may also reflect increased disease burden and greater clinical complexity. Polypharmacy can negatively impact treatment adherence, increase the risk of adverse events and drug interactions, and complicate therapeutic management [30]. In addition, chronic comorbid conditions may contribute to persistent systemic inflammation and functional impairment, potentially influencing TNFi effectiveness and long-term clinical outcomes [31]. Other variables, such as age, time since diagnosis, and hematological biomarkers, did not differentiate the groups, indicating that functionality and treatment response were the primary factors in patient stratification.

In the genetic field, the analysis of polymorphisms in the TNF pathway pointed to potential associations that may influence treatment response. Although most of the SNPs evaluated were not significantly associated with response profiles, the observed association between rs767455 and TNFi response should be interpreted cautiously as a hypothesis-generating finding. Previous studies indicate that polymorphisms in TNF pathway genes may affect therapeutic response, corroborating our findings [11, 31]. However, genetic associations with TNFi response remain heterogeneous across different studies and populations, likely reflecting differences in ethnic background, sample composition, therapeutic protocols, and outcome definitions. Therefore, although isolated polymorphisms may contribute to response variability, their clinical applicability still depends on validation in larger and ethnically diverse cohorts. These findings support the growing interest in pharmacogenomics as a complementary strategy for precision medicine in rheumatoid arthritis [11, 32, 33]. These findings reinforce the importance of future investigations that further explore gene-environment interactions and their influence on therapeutic response.

Although the definition of responder and non-responder groups considered HAQ results, it is worth mentioning that including complementary measures, such as DAS-28, could enhance the robustness of patient classification [13, 14]. The use of additional inflammatory activity indicators would allow for a more comprehensive assessment of the clinical state, strengthening stratification capabilities and guiding therapeutic interventions. In addition, TNFi exposure was modeled descriptively rather than according to cumulative exposure time or exact treatment duration, because patients frequently switched therapies and complete longitudinal treatment timelines were not consistently available in the real-world clinical records. Although this strategy facilitated harmonization of heterogeneous therapeutic histories and clustering interpretation, it may have limited the ability to capture temporal variations in treatment response and exposure-related effects. Future studies incorporating longitudinal exposure metrics may provide a more refined understanding of TNFi response dynamics.

The results of this study suggest the potential need for supervised analyses in future research, which could be applied both to predict treatment response and assess TNFi safety. The incorporation of supervised learning algorithms, combined with expanded datasets, could refine predictive models and, consequently, contribute to precision medicine by optimizing therapeutic strategies and improving clinical outcomes for patients with rheumatoid arthritis [33, 34].

## Conclusion

In summary, this study demonstrated that the response to TNF inhibitors (TNFi) in rheumatoid arthritis is influenced by clinical, behavioral, and genetic factors. Machine learning analysis revealed three distinct patient profiles concerning therapeutic response, physical activity, comorbidities, multiple medication use, and the presence of genetic polymorphisms, such as rs767455. Physical activity and lower BMI were associated with better outcomes, while the presence of comorbidities and genetic polymorphisms may negatively impact TNFi treatment effectiveness. These findings reinforce the importance of treatment personalization and suggest that supervised approaches may enhance the prediction of therapeutic response, contributing to the clinical management of rheumatoid arthritis.

## Supplementary Information

Supplementary Material 1.

## Data Availability

The datasets generated during and analysed during the current study are available from the corresponding author on reasonable request.
