# Supplementary material for: Clustering of rheumatoid arthritis patients: an unsupervised machine learning approach for characterizing the TNFi response
Source: Immunol Res. 2026 Aug 17;74(1):88. doi: 10.1007/s12026-026-09822-x (PMC13481759; doi:10.1007/s12026-026-09822-x)
Supplement: Supplementary file 1 [file 12026_2026_9822_MOESM1_ESM.docx]

**SUPPLEMENTARY TABLES**

**Supplementary Table 1.** Comparison of variables with missing data in the original database with the database containing imputed values

| **Variable** | **Description** | **Variable type** | **Percent NA** | **Test type** | **p_value** |
| --- | --- | --- | --- | --- | --- |
| INEF_TNFi | Effective TNFi: Yes or No | Categoric | 9.2% | Chi-Square | 0.77 |
|  |  |  |  |  |  |
| rs1061624_G | rs1061624 with presence of G allele: Yes or No | Categoric | 7.1% | Chi-Square | 0.60 |
|  |  |  |  |  |  |
| rs1061624_A | rs1061624 with presence of A allele: Yes or No | Categoric | 7.1% | Chi-Square | 1.00 |
|  |  |  |  |  |  |
| rs767455_C | rs767455 with presence of C allele: Yes or No | Categoric | 4.4% | Chi-Square | 0.72 |
|  |  |  |  |  |  |
| rs767455_T | rs767455 with presence of T allele: Yes or No | Categoric | 4.4% | Fisher | 0.19 |
|  |  |  |  |  |  |
| RAM_TNFi | Adverse event related to TNFi: Yes or No | Categoric | 2.7% | Chi-Square | 0.08 |
|  |  |  |  |  |  |
| COLOR_RACE | Self-reported race/ethnicity: White, Multiethnic, or Black | Categoric | 0.7% | Chi-Square | 0.82 |
|  |  |  |  |  |  |
| SMOKING | Smoking status: Non-smoker, Former smoker, or Current smoker | Categoric | 0.3% | Fisher | 0.58 |
|  |  |  |  |  |  |
| VAS | Visual Analogic Scale | Numeric | 6.1% | Wilcoxon | 0.89 |
|  |  |  |  |  |  |
| DIAG_AGE | Age at diagnosis | Numeric | 5.4% | Wilcoxon | 0.88 |
|  |  |  |  |  |  |
| DIAG_TIME | Years since diagnosis | Numeric | 5.4% | Wilcoxon | 0.93 |
|  |  |  |  |  |  |
| HAQ | Health Assessment Quality score | Numeric | 3.1% | Wilcoxon | 1.00 |
|  |  |  |  |  |  |
| BMI | Body Mass Index | Numeric | 2.7% | Wilcoxon | 0.89 |
|  |  |  |  |  |  |
| QNT_CHANGES | Number of biologic treatment changes | Numeric | 2.7% | Wilcoxon | 0.84 |

Variables with missing data (NA) retained in the dataset (< 10% missing data), their descriptions, classification by type, and the respective percentage of missing data. The test used to compare the characteristics of the variables between the datasets and the p-value of this analysis are provided.

**Supplementary Table 2.** Stratification of TNF pathway polymorphic alleles based on response to TNFi treatment in rheumatoid arthritis.

| **Genotype** | **TNFi Responder**  **(n= 129)** | **TNFi Non-Responder**  **(n = 165)** | **Significance**  **(p-value)** |
| --- | --- | --- | --- |
| **rs1800629_A** |  |  |  |
| No | 102 (79.1%) | 116 (70.3%) | 0.12 |
| Yes | 27 (20.9%) | 49 (29.7%) |  |
| **rs1061622_G** |  |  | 1.00 |
| No | 77 (59.7%) | 98 (59.4%) |  |
| Yes | 52 (40.3%) | 67 (40.6%) |  |
| **rs1061622_T** |  |  |  |
| No | 7 (5.4%) | 11 (6.7%) | 0.85 |
| Yes | 122 (94.6%) | 154 (93.3%) |  |
| **rs4149570_A** |  |  |  |
| No | 78 (60.5%) | 96 (58.2%) | 0.78 |
| Yes | 51 (39.5%) | 69 (41.8%) |  |
| **rs4149570_C** |  |  | 0.79 |
| No | 6 (4.7%) | 10 (6.1%) |  |
| Yes | 123 (95.3%) | 155 (93.9%) |  |
| **rs767455_C** |  |  |  |
| No | 33 (25.6%) | 62 (37.6%) | **0.04*** |
| Yes | 96 (74.4%) | 103 (62.4%) |  |
| **rs767455_T** |  |  |  |
| No | 13 (10.1%) | 24 (14.5%) | 0.33 |
| Yes | 116 (89.9%) | 141 (85.5%) |  |
| **rs1061624_G** |  |  |  |
| No | 49 (38%) | 72 (43.6%) | 0.39 |
| Yes | 80 (62%) | 93 (56.4%) |  |
| **rs1061624_A** |  |  |  |
| No | 40 (31%) | 40 (24.2%) | 0.25 |
| Yes | 89 (69%) | 125 (75.8%) |  |

Stratification was conducted based on the response profile to TNFi treatment. All variables represent the presence or absence of specific alleles for the respective polymorphisms. The chi-square test was applied to assess associations between these variables and the treatment response. The value of n represents the subtotal of individuals per group. A p-value < 0.05 was considered statistically significant. *<0.05, **<0.01.

**Supplementary Table 3.** Grouping of categorical variables by hierarchical clustering

| Variable |  | 1 | 2 | | 3 | | X²  (p values) | |  |
| --- | --- | --- | --- | --- | --- | --- | --- | --- | --- |
| **Adverse event associated with TNFi** | No | 147 (79.89%) | | 59 (77.63%) | | 26 (76.47%) | | 0.3 (0.86) | |
|  | Yes | 37 (20.11%) | | 17 (22.37%) | | 8 (23.53%) | |  |  |
|  |  |  | |  | |  | |  | |
| **Use of additional sDMARD** | No | 44 (23.91%) | | 13 (17.11%) | | 13 (38.24%) | | 5.78 (0.055) | |
|  | Yes | 140 (76.09%) | | 63 (82.89%) | | 21 (61.76%) | |  |  |
|  |  |  | |  | |  | |  | |
| **rs1800629_A** | No | 136 (73.91%) | | 56 (73.68%) | | 26 (76.47%) | | 0.11 (0.947) | |
|  | Yes | 48 (26.09%) | | 20 (26.32%) | | 8 (23.53%) | |  |  |
|  |  |  | |  | |  | |  | |
| **rs1061622_G** | No | 108 (58.7%) | | 46 (60.53%) | | 21 (61.76%) | | 0.15 (0.925) | |
|  | Yes | 76 (41.3%) | | 30 (39.47%) | | 13 (38.24%) | |  |  |
|  |  |  | |  | |  | |  | |
| **rs1061622_T** | No | 10 (5.43%) | | 6 (7.89%) | | 2 (5.88%) | | 0.57 (0.752) | |
|  | Yes | 174 (94.57%) | | 70 (92.11%) | | 32 (94.12%) | |  |  |
|  |  |  | |  | |  | |  | |
| **rs4149570_A** | No | 108 (58.7%) | | 46 (60.53%) | | 20 (58.82%) | | 0.08 (0.962) | |
|  | Yes | 76 (41.3%) | | 30 (39.47%) | | 14 (41.18%) | |  |  |
|  |  |  | |  | |  | |  | |
| **rs4149570_C** | No | 10 (5.43%) | | 5 (6.58%) | | 1 (2.94%) | | 0.6 (0.739) | |
|  | Yes | 174 (94.57%) | | 71 (93.42%) | | 33 (97.06%) | |  |  |
|  |  |  | |  | |  | |  | |
| **rs767455_C** | No | 56 (30.43%) | | 27 (35.53%) | | 12 (35.29%) | | 0.79 (0.672) | |
|  | Yes | 128 (69.57%) | | 49 (64.47%) | | 22 (64.71%) | |  |  |
|  |  |  | |  | |  | |  | |
| **rs1061624_G** | No | 73 (39.67%) | | 32 (42.11%) | | 16 (47.06%) | | 0.68 (0.71) | |
|  | Yes | 111 (60.33%) | | 44 (57.89%) | | 18 (52.94%) | |  |  |
|  |  |  | |  | |  | |  | |
| **Smoking** | Former smoker | 37 (20.11%) | | 16 (21.05%) | | 8 (23.53%) | | 0.21 (0.9) | |
|  | Non smoker | 142 (77.17%) | | 56 (73.68%) | | 25 (73.53%) | | 0.47 (0.79) | |
|  | Smoker | 5 (2.72%) | | 4 (5.26%) | | 1 (2.94%) | | 1.09 (0.581) | |
|  |  |  | |  | |  | |  | |
| **Color/Race** | Black | 61 (33.15%) | | 27 (35.53%) | | 11 (32.35%) | | 0.17 (0.92) | |
|  | Multiethnic | 102 (55.43%) | | 35 (46.05%) | | 19 (55.88%) | | 2.02 (0.364) | |
|  | White | 21 (11.41%) | | 14 (18.42%) | | 4 (11.76%) | | 2.37 (0.306) | |

The nonsignificant results of the categorical data indicate the total number of individuals in the cluster and the percentage that this total represents of the study population. X²: chi-square; p: p value. *<0.05, **<0.01,*** <0.001.
